# Synthetic self-adjuvanting glycopeptide cancer vaccines

**DOI:** 10.3389/fchem.2015.00060

**Published:** 2015-10-23

**Authors:** David M. McDonald, Scott N. Byrne, Richard J. Payne

**Affiliations:** ^1^School of Chemistry, The University of SydneySydney, NSW, Australia; ^2^Discipline of Infectious Diseases and Immunology, The University of SydneySydney, NSW, Australia

**Keywords:** glycopeptide, vaccine, tumor-associated carbohydrate antigen, MUC1, antigen, adjuvant

## Abstract

Due to changes in glycosyltransferase expression during oncogenesis, the glycoproteins of cancer cells often carry highly truncated carbohydrate chains compared to those on healthy cells. These glycans are known as tumor-associated carbohydrate antigens (TACAs), and are prime targets for use in vaccines for the prevention and treatment of cancer. Herein, we review the state-of-the-art in targeting the immune system toward tumor-associated glycopeptide antigens *via* synthetic self-adjuvanting vaccines, in which the antigenic and adjuvanting moieties of the vaccines are present in the same molecule. The majority of the self-adjuvanting glycopeptide cancer vaccines reported to date employ antigens from mucin 1, a protein which is highly over-expressed and aberrantly glycosylated in many forms of cancer. The adjuvants used in these vaccines predominantly include lipopeptide- or lipoamino acid-based TLR2 agonists, although studies investigating stimulation of TLR9 and TLR4 are also discussed. Many of these adjuvants are highly lipophilic, and, upon conjugation to antigenic peptides, provide amphiphilic vaccine molecules. The amphiphilic nature of these vaccine constructs can lead to the formation of higher-order structures by vaccines in solution, which are likely to be important for their efficacy *in vivo*.

## Introduction

It is well-established that immunological surveillance is an important method by which cancer is controlled in healthy people. As such, tumor immunology and immunotherapy have emerged as exciting new opportunities for the prevention and treatment of malignancy (Lizée et al., 2013). To date, researchers have sought to use tumor-associated antigens (TAAs) to induce or boost tumor immunity with a view to preventing or treating malignancy. However, as most of the antigens expressed by cancer cells are mutated or over-expressed self-antigens, any attempt to utilize the immune system to clear cancer must first overcome the major hurdle of immunological self-tolerance. Vaccination strategies seek to break tolerance to particular TAAs and induce long-lived adaptive immune responses. As a result, the antigens chosen for the development of a cancer vaccine must be unique to, or highly over-expressed in, cancer cells compared to healthy tissue (Cheever et al., 2009).

**Figure 1:**
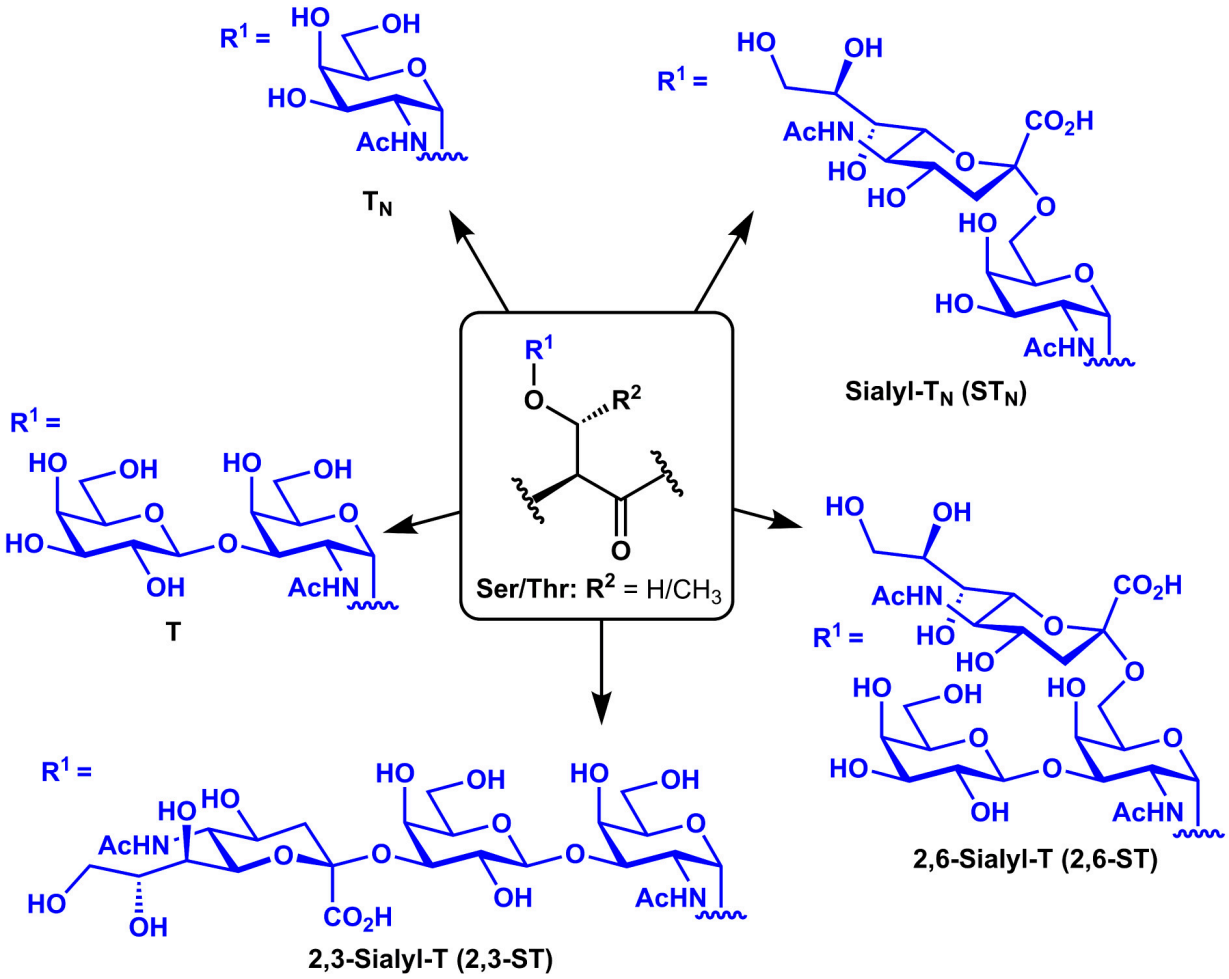
**Structures of common tumor-associated carbohydrate antigens (TACAs)**.

The glycocalyx of healthy epithelial tissues is comprised of densely glycosylated glycoproteins. The glycosylation of these proteins is controlled by the relative concentrations and activities of glycosyltransferases, and mucinous proteins are typically decorated with elongated, branched carbohydrate chains (Shimizu and Yamauchi, 1982). During cancer, changes in the expression of key glycosyltransferases can lead to expression of heavily truncated *O*-linked glycans known

as tumor-associated carbohydrate antigens (TACAs) appended to the peptide backbones of these mucin-type glycoproteins (Meezan et al., 1969; Dennis et al., 1999). Such TACAs include the mono-saccharidyl T_N_ and di-saccharidyl T antigens and their sialylated derivatives (Sialyl-T_N_, 2,3-Sialyl-T, and 2,6-Sialyl-T), which arise from over-expression of sialyltransferases (Figure 1). Several mucin (MUC) glycoproteins have been shown to be overexpressed during cancer (Torres et al., 2012) and have therefore been targeted in cancer vaccines, including MUC1 (Kimura and Finn, 2013), MUC4 (Cai et al., 2015), MUC5AC (Zhu et al., 2009), and MUC16 (Reinartz et al., 2003). MUC1, however, is by far the most commonly studied of these antigens for vaccine development.

### MUC1

MUC1 is a single-pass transmembrane glycoprotein expressed on the apical surface of normal epithelial cells. The extracellular domain of MUC1 contains between 25 and 125 repeats of a 20-amino acid-residue variable number tandem repeat (VNTR) sequence GVTSAPDTRPAPGSTAPPAH. Serine and threonine residues within this repeat are *O*-glycosylated with elongated carbohydrate chains, and each potential glycosylation site can contain glycans *in vivo* (Muller et al., 1997). In many cancers, MUC1 is aberrantly glycosylated and highly overexpressed (Kufe, 2009). Tumor expression of MUC1 is correlated with reduced survival of renal cell carcinoma patients (Fujita et al., 1999) and increased metastatic ability of many cancers (Horm and Schroeder, 2013). For these reasons, MUC1 is considered a promising antigen in the development of effective cancer vaccines. However, the inherent heterogeneity of glycosylation means that chemically uniform MUC1 glycopeptides cannot be isolated, and must instead be chemically synthesized. One approach to generate vaccines incorporating synthetic MUC1 glycopeptides involves the conjugation of MUC1 glycopeptides to carrier proteins such as bovine serum albumin (Cai et al., 2011), keyhole limpet hemocyanin (Adluri et al., 1999), and tetanus toxoid (Kaiser et al., 2009). Such vaccines have been reviewed previously (Gaidzik et al., 2013), and will not be discussed in detail here. Instead, this mini-review will highlight recent research aimed at the development of synthetic self-adjuvanting vaccines.

### Self-adjuvanting vaccines

In order to properly activate antigen-presenting cells (APCs) such as dendritic cells and macrophages toward the priming of cytotoxic and helper T cells (CTLs and T_h_ cells, respectively), vaccines usually incorporate an adjuvant which can stimulate APCs *via* pattern-recognition receptors. Adjuvants are thought to be a necessary feature to make cancer vaccines immunogenic enough to break through immunological self-tolerance to tumor antigens (Mesa and Fernández, 2004). While traditional vaccines are formulated into mixtures of an antigen plus an adjuvant, vaccines in which the two moieties are contained within a single molecule are dubbed self-adjuvanting vaccines. Such vaccines have an advantage over traditional vaccines in that they are taken up by APCs faster (Zhu et al., 2004), while ensuring that the APCs activated by the adjuvant are the same APCs exposed to antigen. Another major advantage is that self-adjuvanting vaccines avoid the use of highly toxic adjuvants such as complete Freund's adjuvant (CFA) while still eliciting potent immune responses (Chua et al., 2014). In experimental models, these approaches have proved beneficial in vaccines against cancer (Le Gal et al., 2002; Liu et al., 2013), infectious diseases (Batzloff et al., 2006; Bettahi et al., 2006), and allergy (Anderson et al., 2014), and have been reviewed previously (BenMohamed et al., 2002; Brown and Jackson, 2005; Moyle and Toth, 2008; Chentoufi et al., 2009).

## Self-adjuvanting glycopeptide cancer vaccines

### Antigens

**Figure 2:**
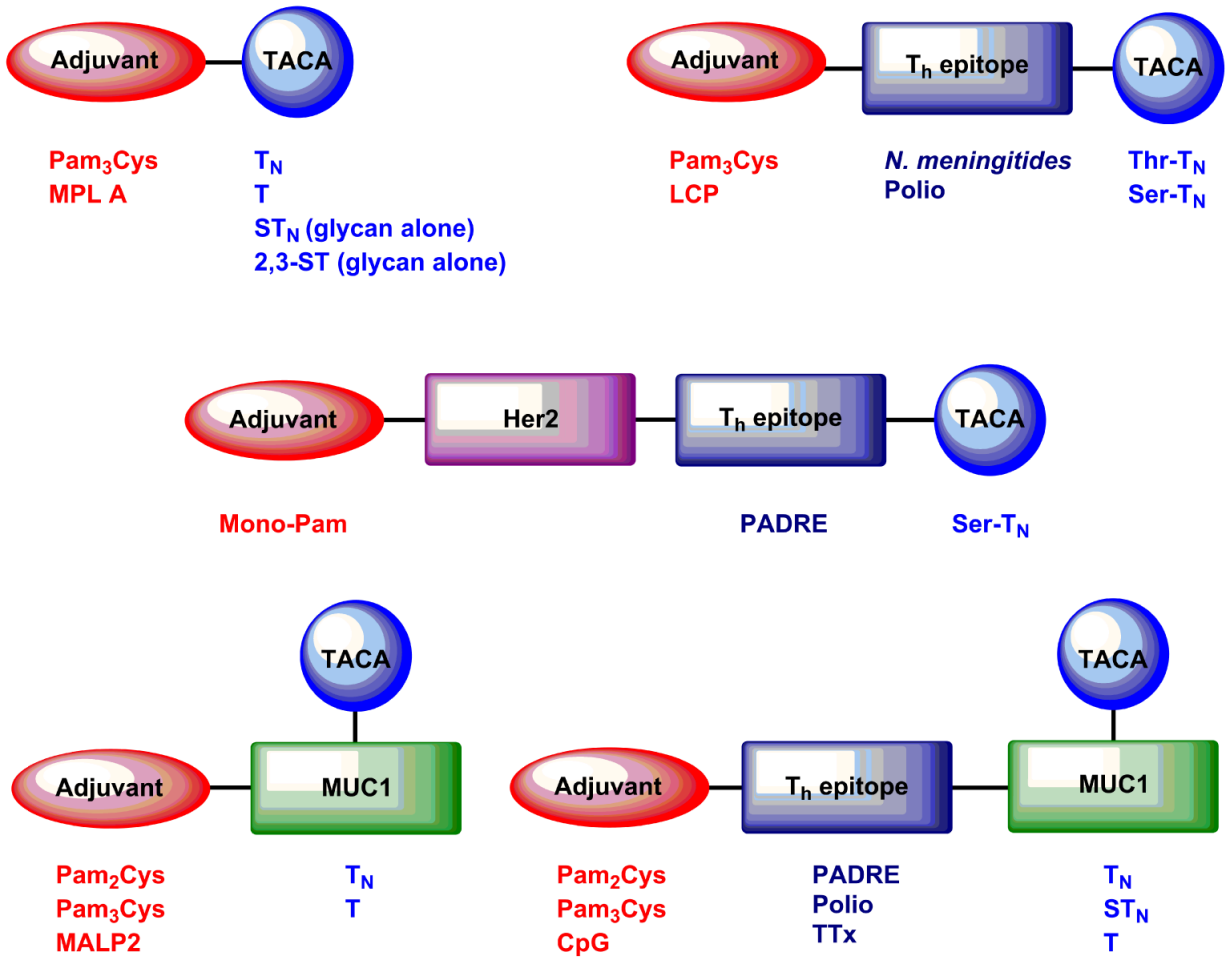
**Examples of self-adjuvanting TACA and glycopeptide cancer vaccines discussed in this mini-review article**.

The studies reviewed here focus on the induction of immunological responses to TACAs and glycopeptides bearing these glycan structures by self-adjuvanting vaccines (Figure 2). Early self-adjuvanting glycopeptide cancer vaccines consisted of clustered TACAs linked *via* spacers to tri-palmitoylated cysteine (Pam_3_Cys) (Toyokuni et al., 1994; Kuduk et al., 1998). These vaccines induced TACA-specific antibodies, predominantly of the IgM isotype. Inclusion of a T_h_ epitope led to the induction of high titers of class-switched IgG antibodies, but did not induce any cellular anti-cancer immunity because no CD8^+^ T cell epitopes were included (Buskas et al., 2005; Abdel-Aal et al., 2012).

Boons and coworkers have reported the use of the immunodominant epitope SAPDT^*^RPAP (Where T^*^ indicates a threonine residue glycosylated with TACAs) conjugated to a polio T_h_ epitope and Pam_3_Cys (Ingale et al., 2007). This 9-mer binds H-2K^b^ in mice (Apostolopoulos et al., 1997a) and contains the human leukocyte antigen (HLA)-A2-binding epitope APDTRPA (Apostolopoulos et al., 1997b). Additionally, the peptide contains the immunodominant MUC1 B cell epitope PDT^*^RP. When formulated into liposomes and injected into mice, these vaccines gave rise to high titers of MUC1-specific antibodies, as well as up-regulation of tumor necrosis factor (TNF), CD83, and the co-stimulatory molecules CD80 and CD86. Importantly, the authors have recently demonstrated induction of CTL responses and inhibition of mouse mammary tumor growth in response to these vaccines (Lakshminarayanan et al., 2012).

We and others have used full 20-amino acid residue MUC1 VNTR peptides in self-adjuvanting vaccines. Kunz and coworkers reported the induction of MUC1-specific antibodies in response to vaccination with CFA and a Pam_3_Cys-MUC1 VNTR conjugate containing a full copy of the VNTR (PAHGVT^*^SAPDTRPAPGSTAP) glycosylated with T_N_, T, or 2,6-ST at a single Thr residue within the VNTR (indicated by asterisk) (Kaiser et al., 2010). We incorporated a per-glycosylated MUC1 epitope (GVT^*^S^*^APDT^*^RPAPGS^*^T^*^APPAH), whereby all possible glycosylation sites were occupied with TACAs (indicated by asterisks), in order to maximize the potential recognition of glycopeptide tumor-associated epitopes (Wilkinson et al., 2011). Vaccines containing per-glycosylated MUC1 antigens, when injected in PBS without external adjuvant or formulation into liposomes, induced high titers of MUC1 antibodies capable of recognizing MCF-7 breast cancer cells. CD8^+^ T cell responses were not observed, possibly due to over-glycosylation of the VNTR backbone leading to reduced presentation of CD8^+^ T cell epitopes. Li and coworkers used vaccines containing full-length MUC1 VNTR epitopes glycosylated variably at a Ser and/or Thr residue within the VNTR (HGVTSAPDT^*^RPAPGS^*^TAPPA) to induce IgG antibodies capable of recognizing tumor cells (Cai et al., 2014). However, while these were capable of inducing antibody-dependent cell-mediated cytotoxicity (ADCC), no CD8^+^ T cell responses were reported for these vaccines. It is worth noting that different MUC1 VNTR sequences were used in each of these studies, and each vaccine consequently contained different possible T and B cell antigens. In the future, we envisage that use of longer VNTR peptides containing overlapping repeats will be important to cover the full range of epitopes in the VNTR sequence.

### Multi-antigenic vaccines

A necessary feature of self-adjuvanting vaccines is that they target the immune system to particular, well-defined molecular epitopes such as tumor-associated glycoforms of the MUC1 VNTR. This can be problematic, since mutation or down-regulation of tumor antigens can lead to tumor escape and growth even in the presence of a robust antigen-specific anti-tumor immune response (Kim et al., 1975; Stackpole et al., 1980). One solution is to utilize long peptides containing multiple short TAA epitopes (Slingluff, 2011), although such an approach has yet to be widely adopted for self-adjuvanting glycopeptide cancer vaccines. BenMohamed and colleagues showed reduced tumor burden and increased survival in response to self-adjuvanting vaccines containing four copies of the T_N_ antigen in concert with a CD8 epitope from the breast cancer antigen human epidermal growth factor receptor 2 (Her2) and the pan HLA DR-binding Epitope or “PADRE” (Renaudet et al., 2010). Protection from murine breast cancer was associated with both generations of TACA-specific antibodies and Her2-specific, Interferon (IFN)-γ-secreting CD8^+^ T cells.

### Adjuvants

Most self-adjuvanting cancer vaccines have utilized lipopeptide-derived toll-like receptor (TLR)-2 agonists as the adjuvant. Tri- and di-palmitoylated cysteine (Pam_3_Cys and Pam_2_Cys) agonize TLR1/2 heterodimers and TLR2/6 heterodimers, respectively, leading to signaling *via* the NF-κB pathway and APC activation. Monopalmitoylated peptides (Bettahi et al., 2009) and lipoamino acids (Abdel-Aal et al., 2012) have also been investigated, which both signal *via* TLR2.

We recently reported the synthesis and evaluation of self-adjuvanting vaccines containing macrophage-activating lipopeptide 2 (MALP2), which contains Pam_2_Cys as part of the structure (McDonald et al., 2014). Interestingly, we observed very high titers of class-switched, MUC1-specific antibodies in response to MALP2-containing vaccines in the absence of a helper T cell epitope and the absence of a measurable T_h_ cell response, indicative of a T cell-independent humoral response.

Boons and coworkers compared MUC1 conjugate vaccines containing unmethylated CpG nucleotides, a TLR9 agonist, to Pam_3_CysSKKK (Abdel-Aal et al., 2014). They found that, although vaccines containing CpG could induce MUC1-specific antibodies, those antibodies were less efficient at inducing ADCC against cancer cell lines than antibodies raised against Pam_3_Cys-containing vaccines. Furthermore, Pam_3_Cys-containing vaccines led to a reduction of mouse mammary tumor burden and induced CTLs capable of killing MUC1-overexpressing cell lines, neither of which was observed after treatment with CpG-containing vaccines. The authors hypothesized that the increased efficacy of Pam_3_Cys-containing vaccines over CpG-containing vaccines might be due to the ability of TLR2 agonists to reduce the function of regulatory T cells.

Monophosphoryl lipid A (MPLA) is a TLR4 agonist derived from bacterial lipopolysaccharide. Guo and coworkers reported the synthesis and evaluation of self-adjuvanting vaccines consisting of MPLA conjugated to the 2,3-ST (Wang et al., 2012) and ST_N_ (Zhou et al., 2014) TACAs. The MPLA conjugates induced TACA-specific IgG antibodies capable of recognizing cancer cells.

A few studies have investigated the interaction between self-adjuvanting vaccines and traditional experimental vaccines. The adjuvant QS-21 in combination with a Pam_3_Cys-containing vaccine was shown to lead to T_h_2 polarization compared to the vaccine alone, but induced similar levels of MUC1-specific IgG antibodies (Lakshminarayanan et al., 2012). Surprisingly, in some instances the inclusion of CFA actually led to inhibition of self-adjuvanting vaccine immunogenicity (Huang et al., 2012; Cai et al., 2013), while in others CFA inclusion led to increased antibody production (Abdel-Aal et al., 2012). Similarly, co-injection of MPLA conjugates with Titermax gold completely abrogated anti-TACA immunity (Wang et al., 2012; Zhou et al., 2014).

### Synthesis

Generally, chemical approaches for the synthesis of tumor antigens containing TACAs have involved incorporation of suitably protected glycosylserine/threonine residues into nascent peptides *via* Fmoc-strategy solid phase peptide synthesis (SPPS), with deprotection of the carbohydrates after cleavage of the crude glycopeptide from resin. Various syntheses of T_N_, ST_N_, T, and ST TACA-derived amino acids for incorporation into SPPS have been reported to date (Brocke and Kunz, 2002; Dziadek and Kunz, 2004; Wilkinson et al., 2010; Corcilius and Payne, 2013; Wilson and Danishefsky, 2013). It should be noted that incorporation of TACAs has also been achieved through chemoenzymatic means (Bézay et al., 2001; Sorensen et al., 2006; Bello et al., 2014).

The most challenging step in the development of self-adjuvanting vaccines is often the conjugation of (glyco)peptide antigens to lipopeptide adjuvants. The direct incorporation of lipidated adjuvants such as Pam_2_Cys and Pam_3_Cys into short peptides *via* SPPS works well (Metzger et al., 1991), including through the use of microwave-assisted SPPS (Thompson et al., 2015). However, longer self-adjuvanting vaccine constructs often produce complex mixtures which are difficult to purify. We have reported the efficient synthesis of such vaccines *via* the fragment condensation of suitably protected (glyco)peptide fragments activated at the C-termini as pentafluorophenyl esters (Wilkinson et al., 2010). This approach generated the desired self-adjuvanting vaccine candidates in good to excellent yields, but is incompatible with unprotected lysine side-chains (which could undergo unwanted fragment condensation reactions), necessitating the purification of protected lipopeptide fragments using normal-phase HPLC. Li and coworkers utilized a side-chain deprotected, iodoacetylated Pam_3_Cys-Ser-(Lys)_5_ to conjugate unprotected Pam_3_Cys to MUC1 glycopeptide 20-mers *via* a thioether linkage in moderate to good yields (Cai et al., 2013).

Boons and coworkers have reported the synthesis of vaccine constructs containing Pam_3_Cys *via* liposome-mediated native chemical ligation (NCL) (Ingale et al., 2006). The use of dodecylphosphocholine liposomes was necessitated by the insolubility of Pam_3_Cys-containing peptides in ligation buffer. While this approach has the benefit of chemoselectivity, it requires formulation of each ligation fragment into liposomes, followed by HPLC after each ligation to separate the ligation products from the liposomal components.

### Higher-order structures

Another major factor that underlies choice of adjuvant is the higher-order structure of the vaccine constructs. Peptide amphiphiles have long been known to form ordered structures in solution, and it has been demonstrated that MUC1 glycolipopeptide-based self-adjuvanting vaccines can form stable, ordered nanoparticles in solution (Wilkinson et al., 2012). The long lipid chains of TLR2 agonists support the formation of such structures through hydrophobic interactions. Moreover, the multivalent presentation of adjuvant and antigen moieties that results from such structures is likely to be important for the activation of APCs (Bachmann and Jennings, 2010; Oyewumi et al., 2010) and possibly underpins the differences in vaccine efficacy between various classes of adjuvant.

The location of the lipid-containing adjuvant within self-adjuvanting vaccines has ramifications for the resultant immune response. BenMohamed and coworkers found that a linear glycolipopeptide led to increased IFN-γ production by CD8^+^ T cells and inhibition of tumor growth compared to a branched analog (Renaudet et al., 2010). The authors demonstrated *in vitro* that this was associated with a difference in uptake by DCs. Furthermore, the authors showed that the two vaccines were subject to different cross-presentation pathways, which may explain the difference in DC and T cell activation observed. Similarly, Toth and coworkers found significantly higher T_N_-specific antibodies in response to a linear vaccine construct than a branched construct (Abdel-Aal et al., 2012). In a more recent study (Eskandari et al., 2015), the same group found that the location of the TLR-2 agonist in the middle or at the terminus of similar constructs containing ovalbumin model antigens controlled the physical properties of the self-assembled particles observed in solution.

Li and colleagues synthesized a range of vaccine constructs containing full-length MUC1 VNTR glycopeptides conjugated to the Q11 peptide aggregation sequence (Huang et al., 2012). The resulting constructs formed well-defined fibrils over 200 nm in length, irrespective of glycosylation pattern. These constructs were able to induce MUC1-specific, class-switched antibodies in the absence of a canonical adjuvant, and were capable of inducing complement-dependent lysis of MCF-7 breast cancer cells, while T cell responses to the vaccines were not reported. IgG antibody titers induced by these constructs were 1–2 orders of magnitude lower than those typically seen for vaccines containing a TLR2 agonist (Ingale et al., 2007; Wilkinson et al., 2011; Cai et al., 2013). Nonetheless, it remains an excellent example of the importance of higher-order structure and multivalent antigen presentation to the immune response elicited by conjugate vaccines.

It is clear from these studies that the formation of higher-order structures by self-adjuvanting vaccines plays an important role in their immunogenicity. To date, however, the relationships between vaccine primary structure, higher-order organization in solution, and resulting immunological response are poorly understood, and should serve as an important subject for study in the future to better understand immune stimulation by self-adjuvanting constructs.

## Conclusions and outlook

Self-adjuvanting glycopeptide cancer vaccines are a promising avenue for the prevention and treatment of human cancers. Here, we have briefly highlighted selected literature examples that showcase the design, synthesis and immunological evaluation of such vaccines.

The antigens incorporated into self-adjuvanting cancer vaccines range from clustered copies of the T_N_ antigen to truncated MUC1 B cell epitopes to full copies of glycosylated MUC1 VNTR. Although each of these antigens has been shown to generate potent humoral immune responses, few studies have reported induction of T cell-mediated immunity toward MUC1-expressing cells. It will be important to dissect the relative contribution of these two types of adaptive immune responses in both the prophylactic and therapeutic treatment of MUC1-expressing tumors. A major goal moving forward should be to maximize the anti-tumor CTL response, which appears to be particularly important in achieving this objective. The field would benefit from a comprehensive analysis of how the location and identity of MUC1 TACAs influences anti-tumor immune responses in order to design vaccines which produce *optimal humoral and cell-mediated immunity*. Furthermore, the development of multi-antigenic vaccines could engage both the humoral and cellular arms of the immune system while diversifying the range of TAAs targeted.

Most of the studies reviewed here utilized TLR2 agonists, including tri- di- and mono-palmitoylated cysteine, lipoamino acids, and MALP2. All of these adjuvants in isolation have shown efficacy for the induction of immune responses to conjugated TAAs, but very few studies have compared the effects of different conjugated adjuvants. In one such study, Pam_3_Cys was shown to be superior to CpG, a TLR9 agonist, in similar conjugate vaccines containing adjuvant, a MUC1 VNTR epitope and a helper T cell epitope.

The use of lipidated amino acids in self-adjuvanting vaccines leads to highly amphiphilic molecules, which can form higher-order structures in solution. Such molecular organization can give rise to multimeric antigen presentation, and contribute to vaccine efficacy. A number of solutions have arisen to combat the purification difficulties which can also arise from such amphiphilic molecules, but a general method for the simple conjugation and purification of glycolipopeptides from purified, unprotected fragments would be invaluable for further development of self-adjuvanting vaccines.

In conclusion, the studies reviewed here collectively demonstrate that self-adjuvanting glycopeptide cancer vaccines are promising targets for the prevention and treatment of cancer. Improved synthetic routes and new methodologies for the production of glycolipopeptides have made and will continue to make self-adjuvanting vaccines synthetically feasible. In concert with this, *in vivo* studies investigating the efficacy of different combinations of adjuvants and antigens have led to the production of conjugate vaccines capable of eliciting high-titer antibodies and potent CTL responses. In the future, we envisage these studies will inform the translation of self-adjuvanting glycopeptide cancer vaccines into the clinic, while providing the basis for novel experimental therapies incorporating new cancer antigens.

## Funding

The synthetic cancer vaccine research program in our laboratories is supported by a Cure Cancer Australia Priority-driven Collaborative Cancer Research Grant (1049757). DM is supported by an Australian Postgraduate Award and by a John A. Lamberton Research Scholarship.

### Conflict of interest statement

The authors declare that the research was conducted in the absence of any commercial or financial relationships that could be construed as a potential conflict of interest.
